# Economic effects of next-generation sequencing diagnostics in unspecific sepsis patients – a budget impact analysis from the healthcare providers’ perspective in Germany

**DOI:** 10.1007/s10096-024-04940-6

**Published:** 2024-09-24

**Authors:** Anne Wenzel, Johanna Röder, Tabea Poos, Fabian Dusse, Florian Kron

**Affiliations:** 1 VITIS Healthcare Group, Cologne, Germany; 2 Department of Anaesthesiology and Intensive Care Medicine, Faculty of Medicine, University Hospital of Cologne, University of Cologne, Cologne, Germany; 3 FOM University of Applied Sciences, Essen, Germany

**Keywords:** Sepsis, Next-generation sequencing, Budget impact analysis, Health economics, Healthcare provider

## Abstract

**Purpose:**

Next-generation sequencing (NGS) tools have clinical advantages over blood culture but are more expensive. This study assesses the budget impact and break-even point of NGS testing costs from a healthcare provider’s perspective in Germany.

**Methods:**

The budget impact was calculated based on aggregated data of German post-operative surgery cases. Simulated cost savings were calculated based on a simulated reduction in hospital length of stay (LOS) of four or eight days with a positivity rate of 71% and compared to the costs of one (scenario A) or two tests (scenario B) per case. Furthermore, the break-even point of the cost of two tests compared to saved costs through shortened LOS was conducted.

**Results:**

For 9,450 cases, an average budget impact for scenario A and scenario B of €1,290.41 [95% CI €1,119.64 – €1,461.19] and - €208.59 [95% CI - €379.36 – - €37.81] was identified for gastrointestinal and kidney surgery cases, and €1,355.58 [95% CI €1,049.62 – €1,661.55] and €18.72 [95% CI - €324.69 – €287.24] for vascular artery surgery cases, respectively. The break-even analysis showed that using two tests per case could achieve a minimum positive contribution margin with an average of 1.9 tests per case across the study population.

**Conclusion:**

The results revealed a positive budget impact for one NGS test and a slightly negative budget impact for two NGS tests per case. Findings suggest that largest cost savings are generated for more severe cases and are highly dependent on the patient population.

## Introduction

Sepsis is a serious medical emergency caused by various pathogens and infections. The dysregulated host response to causative infections makes sepsis a life-threatening condition with severe organ dysfunction and mortality rates of 25–50% [1, 2]. Worldwide, almost 49 million cases and 11 million sepsis-related deaths were reported in 2017 [3], making sepsis one of the most prevalent diseases and leading cause of mortality. Globally, sepsis accounts for approximately 20% of deaths [4].

Although sepsis-related mortality rates have decreased in Germany in recent years, the incidence rates are still rising [5]. In 2010, an estimated 108 patients per 100,000 population were diagnosed with sepsis, whereas the incidence increased to 158 per 100,000 population in 2015 [6]. Rose et al. (2021) even reported an incidence of 178 per 100,000 [7]. Moreover, incidence rates are likely to be underestimated since data sources consist of variable routine and billing data relying on complete coding [5]. The rise in incidence can be attributed to the aging demographic with a higher prevalence of comorbidities and advanced treatment options weakening the immune system [7, 8]. Studies have shown that surgical patients admitted to intensive care units (ICU) have higher risks of developing sepsis [9].

Sepsis patients represent a high economic burden for healthcare systems and budgets due to time-consuming and resource-intensive treatments during hospitalization and subsequent care. With each sepsis-associated case incurring expenditures of approximately €27,500, as reported by the German Federal Office for Social Security, annual costs of in- and outpatient sepsis patients cumulated to €7.7 billion in Germany in 2013 [10]. However, studies show that early recognition and treatment of sepsis are associated with improved patient outcomes. Rapid detection of sepsis-causing pathogens followed by early administration of medical therapy is linked to lower morbidity and mortality rates [11]. For every hour of delay in antibiotic therapy, sepsis-related mortality increased by approximately four percent, highlighting the need for early administration of effective antibiotics as a key part of sepsis management [12].

Conventional diagnostic techniques rely on culture-based procedures [13], which typically demand several days to detect a limited range of pathogens and are associated with low sensitivity and specificity [14, 15]. Several studies suggest a positivity rate between 10% and 30% for a single blood culture test [16, 17]. Polymerase chain reaction (PCR)-based methods offer a faster approach to pathogen diagnostics but are limited in the number of pathogens they can detect [18]. In contrast, novel technologies, such as the pathogen test DISQVER®, combine next-generation sequencing (NGS) with bioinformatic algorithms to ensure more reliable and faster pathogen detection than blood culture diagnostics. Based on a single blood sample, cell-free DNA is sequenced and matched against a clinical genome database to identify microbes and pathogens. Compared to traditional diagnostics, this approach identifies potential pathogens up to three times more frequently within 24 hours. Besides the medical advantages for patients, NGS diagnostics were also found to decrease the hospital length of stay (LOS) in sepsis patients due to faster diagnosis and timely onset of appropriate treatment, saving hospitals’ financial resources.

Thus, this study aims to calculate the budget impact of NGS compared to common blood culture diagnostics from a healthcare provider/hospital perspective in Germany. The focus lies on post-operative patients with non-specific sepsis, who may benefit from faster pathogen detection resulting in a lower total length of hospitalization. Furthermore, the break-even point of test costs of NGS is analysed.

## Methods

The budget impact analysis is performed from the healthcare provider perspective for the year 2022. The model assumptions are based on the following findings of Grumaz et al. (2019) [15]:

1. Costs for NGS pathogen tests are additional expenses on behalf of the hospital not reflected in the remuneration system. Furthermore, the aG-DRG remuneration fee is considered to represent the costs of the cases.
2. NGS alone can potentially reduce the LOS of postoperative patients with susceptible sepsis by eight days on the general ward (GW) and by four days in the ICU compared to the use of blood culture diagnostics alone.
3. NGS was found to have a positivity rate of 71% resulting in 71% of all performed NGS tests providing a positive test result.

The budget impact of the pathogen test DISQVER® instead of blood culture was assessed by comparing additional diagnostic costs with indirect savings caused by a simulated reduction in LOS. Given the fact that German hospitals receive flat rates per case for remuneration, a reduction in LOS is generally associated with lower daily costs for hospitals. The simulation was performed for one diagnostic test per case in scenario A, and two tests per case in scenario B as recommended as a minimum number in the S3 guideline sepsis – prevention, diagnosis, therapy, and follow-up [25]. According to the manufacturer Noscendo GmbH, one DISQVER® pathogen test adds diagnostic costs of €1,499.

### Data source and in- and exclusion criteria

The data set consists of public data which hospitals report quarterly to the Institute for Hospital Remuneration System (InEK), accessible via the InEK Data Browser [19]. The database provides anonymous, aggregated case information of all German hospitals according to the outsourced German Diagnosis Related Groups system (aG-DRG). For reasons of data protection, cases with a prevalence of less than four are not displayed. The latest complete annual data from January 1st until December 31st, 2022, was extracted and analysed.

To obtain case data for post-operative patients with unspecific sepsis, respective diagnostic (ICD code) and procedural codes (OPS code) were identified according to the International Statistical Classification of Diseases 10 German Modification (ICD-10-GM) [20] and the German procedure classification system [21]. Patients with specific sepsis (ICD code A41.0 – A41.8) were excluded from the study since sepsis-causing pathogens had already been identified. Hence, only cases with unspecific sepsis (ICD Code A41.9, ‘Bacterial infection, not further specified’) were considered. Blood culture tests are not identifiable in the aggregated data since the test does not have a specific operations and procedure code (OPS-Code).

The primary study population in Grumaz et al. (2019) comprised postoperative patients who underwent surgery on one or several of the following categories: kidney, liver, pancreas, gastrointestinal tract, and vascular artery surgery [15]. Thus, the patient characteristics in the current study are modelled accordingly. The respective OPS codes are listed in Table 1 below and categorized into two groups: ‘gastrointestinal tract including kidney surgeries’(GIT) and ‘vascular artery surgery’(VAS). Cases were limited to patients aged between 60–64 and 65–74 years to match the study population of Grumaz et al. who reported their effects for a study population aged between 60.5–74 years [15]. Both male and female cases were included.

**Table 1:** Inclusion criteria

Category	Subcategory	Coding information
Diagnosis	Bacterial infection, not further specified	ICD Code A41.9
Age, years	60-74	
Sex	Female and male	
Procedures	Gastrointestinal tract	OPS code groups
	Kidney surgery	5-550 – 5-559.y
	Liver surgery	5-500 – 5-509.x
	Pancreas surgery	5-520 – 5-529.y
	Esophagus surgery	5-420.0 - 5-42a.2
	Incision, excision, and resection on the stomach	5-430.0 - 5-439
	Other organs in the stomach	5-444 – 5-44a.3
	Incision, excision, resection, and anastomosis on small and large bowels	5-450 – 5-459.y
	Other surgery on small and large bowels	5-470 – 5-479.y
	Appendix surgery	5-470 – 5-479.y
	Rectal surgery	5-480 – 5-489.y
	Anus surgery	5-490 – 5-499.y
	Gallbladder and bile duct surgery	5-510 – 5-519.y
	Closure of abdominal hernias	5-530 – 5-539-y
	Other surgery in the abdominal area	5-540 – 5-549.y
	Vascular artery surgery	OPS code groups
	Incision, embolectomy, and thrombectomy of blood vessels	5-380 – 5-38b.y
	Other operations on blood vessels and additional information on blood vessel surgery	5-390 – 5-39a.4
	Surgery on coronary vessels	5-360 – 5-369.y
	Incision, excision, destruction, and obstruction of intracranial blood vessels	5-025 – 5-025.y
	Reconstruction of intracranial blood vessels	5-026 – 5-026.y
	Application of bypass and transposition of intracranial blood vessels	5-027 – 5-027.y
	Surgery on intraspinal blood vessels	5-037 – 5-037.y
	(Percutaneous) transluminal vessel intervention	8-836 – 8-83d.a

### Data collection

Data was retrieved from InEK Data Browser in three consecutive searches per OPS code group [19]. Firstly, the main diagnosis was set as unspecific sepsis (A41.9) AND OPS codes were set for kidney surgery (5-550–5-559.y) AND age restriction was set between 60–64 and 65–74. Secondly, secondary diagnosis was set as unspecific sepsis (A41.9) AND OPS Codes were set for kidney surgery (5-550–5-559.y) AND age restriction was set between 60–64 and 65–74. Thirdly, the search was conducted analogously with unspecific sepsis as main AND secondary diagnosis.

The LOS, number of cases and standard deviation of LOS of cases with the same aG-DRG from the first and second searches were then cumulated by weighted addition. The outcome of the third search represents duplicates that were subtracted accordingly from the cumulated data in a weighted manner. This process was repeated for each OPS code group. The results entail the aG-DRG, its description, number of cases, average LOS, and standard deviation of LOS [19].

The indication whether an aG-DRG describes a GW or ICU case was retrieved through the InEK extreme cost report 2021 [22]. Furthermore, resulting aG-DRGs from InEK Data Browser were linked to the aG-DRG flat rate catalogue to complement following information: valuation ratio for the main department, average LOS, lower and upper limit of LOS: first day with deduction, and first day with surcharge [23]. Cases with an aG-DRG stating a one-day hospital stay, death, an average LOS shorter than 8 days or shorter than the average LOS set in the aG-DRG flat rate catalogue were excluded from the analysis.

### Model design

Cases received based on the OPS codes represented in Table 1 were divided into subgroups GIT and VAS by type of surgery. The groups were further categorized into ICU and GW to allow for comparison by type of surgery and hospital ward. To calculate the budget impact and break-even point of NGS compared to the status quo of blood culture diagnostics, the average aG-DRG remuneration per case after the deduction of additional test costs was calculated. The aG-DRG remuneration thereby indicates the average treatment costs for the hospital based on the average LOS of aggregated aG-DRG cases clustered by OPS code groups listed in Table 1. The aG-DRG result in average remunerations which are assumed to reflect treatment costs for the purpose of this study. The model was constructed in Microsoft Excel Version 2403. The simulated reduced LOS was calculated by subtracting assumed reductions in subgroups GIT/ICU and VAS/ICU (four days) and subgroups GIT/GW and VAS/GW (eight days), respectively. The modelling of cost savings was based on the comparison of the LOS of the data set with the simulated reduced LOS to determine the simulated case mix as well as simulated daily and total remuneration savings per aG-DRG. A reduction in LOS in days was assessed as cost savings for the hospital in proportion to the aG-DRG remuneration.

### Break-even analysis

Firstly, the break-even analysis ‘test cost’ was performed for the entire study population to determine a price for NGS tests at which additional costs per case equal additional indirect revenues of the hospitals by reduced LOS. Each patient with suspected sepsis was assumed to receive two NGS tests per hospitalization. Secondly, the break-even analysis ‘test number’ was conducted for the entire study population as well as per group GIT and VAS to determine the number of feasible tests per case for a balanced return with neither cost savings nor additional costs on the premise of €1,499 per test.

### Statistical analysis

The confidence interval of 95% was computed for subgroups (GIT/ICU, GIT/GW, VAS/ICU, VAS/GW) for scenario A (one test) and scenario B (two tests) to account for uncertainty regarding the mean cost savings. Furthermore, to account for parameter uncertainty, a deterministic sensitivity analysis of +/- 20 percentage points to the positivity rate of 71% was performed. The impact of positivity rates of 51% and 91% on the mean cost savings was evaluated for one and two tests.

## Results

### Study population

**Fig. 1:**
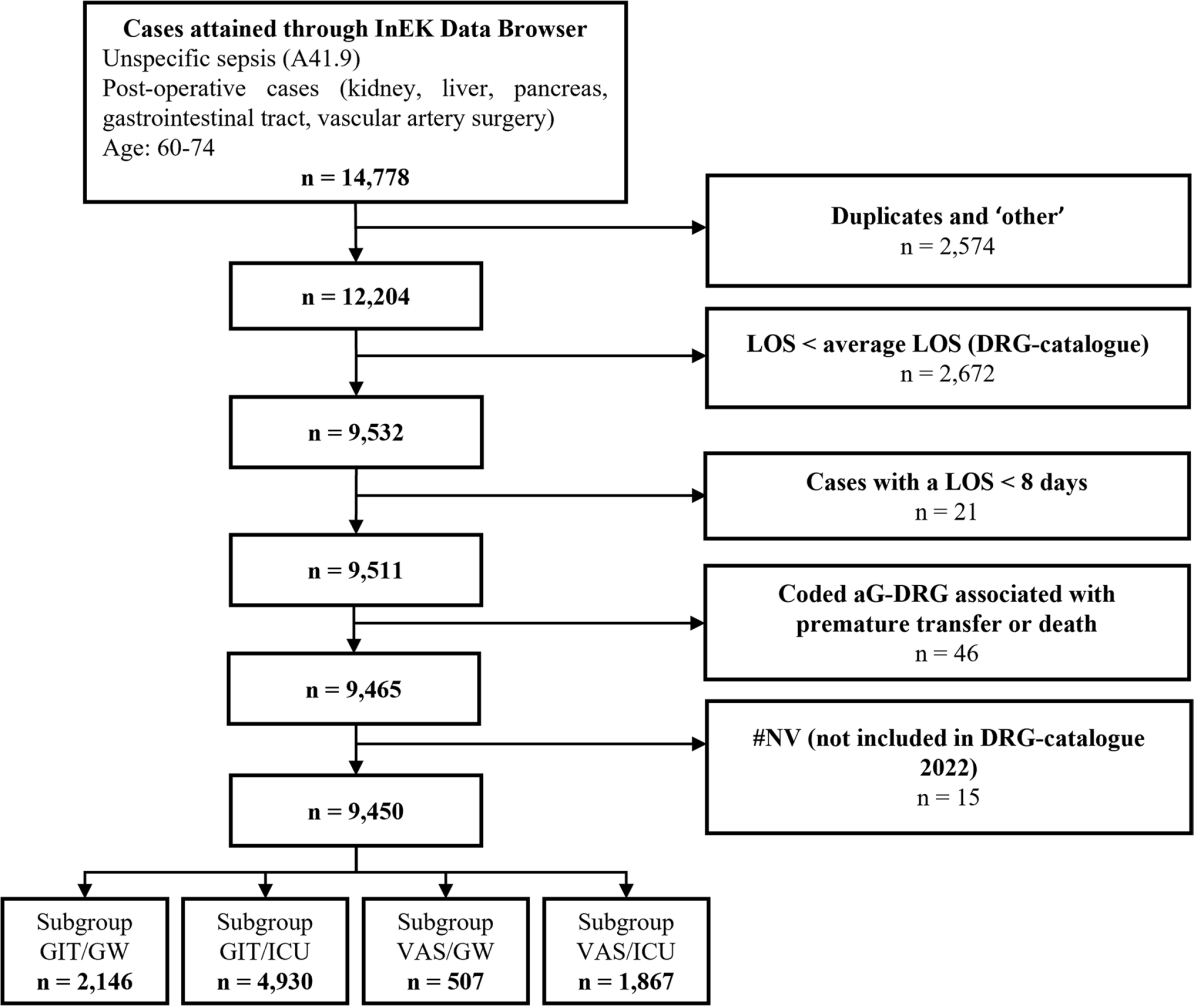
Flow chart of data retrieval. aG-DRG: outsourced German Diagnosis Related Groups, LOS: length of stay

After removing duplicates, 12,204 cases with unspecific sepsis were identified. To be in accordance with the original study by Grumaz et al. (2019) which based the assumptions of the LOS reduction on severe cases with longer than average LOS, 2,672 cases were excluded as LOS was below the average LOS according to the aG-DRG-catalogue 2022. Further 46 cases were excluded by a coded aG-DRG associated with patients’ premature transfer or death. Additionally, 21 cases of the GW were excluded as the LOS was less than eight days and below the assumed reduction for the GW. Another 15 cases were excluded since the aG-DRGs were not listed in the main section of the aG-DRG catalogue 2022. Finally, a total of 9,450 cases were included for analysis. The selection strategy is presented in Fig. 1 below.

More than two-thirds of all cases were assigned to group GIT (7,076), whereas the remaining 2,374 cases were categorised as VAS. Furthermore, ICU-related cases accounted for 71.93% (6,797 cases) of the study population. The remaining 28.07% of the patients (2,653 cases) were treated on the GW.

Case characteristics showed an average LOS of 43.22 days and an average case-mix-index (CMI) of 10.453 across the entire study population, representing the severity level and thus providing information on the relative economic resource consumption. The average LOS for ICU was 49.37 days and the average CMI was 12.833. GW cases had an average LOS of 27.83 days and an average CMI of 4.356. Group-specific characteristics are shown in Table 2.

**Table 2:** Patient characteristics

	Number of cases	Cumulated LOS	Average LOS	Case mix	CMI
Entire study population	9,450	408,444	43.22	98,780	10.453
Study population (ICU)	6,797	335,536	49.37	87,224	12.833
Study population (GW)	2,653	73,832	27.83	11,556	4.356
Group 1 (GIT)	7,076	285,390	40.33	66,694	9.425
Subgroup GIT/GW	2,146	56,431	26.30	8,998	4.193
Subgroup GIT/ICU	4,930	228,959	46.44	57,696	11.703
Group 2 (VAS)	2,374	123,978	52.22	32,085	13.515
Subgroup VAS/GW	507	17,401	34.32	2,558	5.045
Subgroup VAS/ICU	1,867	106,577	57.08	29,528	15.816

*GIT* gastrointestinal tract, *GW* general ward, *ICU* intensive care unit, *VAS* vascular artery surgery

### Comparison GIT and VAS cases

A positive budget impact from a hospital’s perspective through cost savings by reduced LOS was identified for both GIT and VAS for scenario A. Average savings of €1,290.41 [95% CI: €1,119.64 – €1,461.19] for GIT and €1,355.58 [95% CI: €1,049.62 – €1,661.55] for VAS were simulated for one assumed NGS test. Simulated aG-DRG remuneration ranged from - €495.52 to €3,298.57 for GIT and from - € 164.48 to €6,965.16 for VAS cases, respectively. In contrast, no positive budget impact was found for both groups in scenario B. Additional costs of €208.59 [95% CI: - €379.36 – - €37.81] for GIT and €18.72 [95% CI: - €324,69 – €287,24] for VAS result from two NGS tests per case. Results of the subgroup analysis are shown in Table 3.

**Table 3:** Subgroup analysis of potential savings for scenario A (1 test) and B (2 tests)

	Scenario A (1 test)		Scenario B (2 tests)	
	Group 1 (GIT)	Group 2 (VAS)	Group 1 (GIT)	Group 2 (VAS)
*GW and ICU*				
Mean [95% CI]	€1,290.41 [€1,119.64 – €1,461.19]	€1,355.58 [€1,049.62 – €1,661.55]	- €208.59 [- €379.36 – - €37.81]	- €18.72 [- €324,69 – €287,24]
Median (Range)	€1,238.22 (- €495.52 - €3,298.57)	€1,333.00 (- € 164.48 - €6,965.16)	- €260.78 (- €1,994.52 - €1,799.57)	- €166.00 (- €1,663.48 - €5,466.16)
*GW*				
Mean [95% CI]	€1,512.98 [€1,261.29 – €1,764.67])	€1,611.28 [€1,016.58 – €2,205.98]	€13.98 [- €237.71 – €265.67]	€112.28 [- €482.42 – €706.98]
Median (Range)	€1,503.62 (- €495.52 - €3,298.57)	€1,502.58 (- €164.48 - €6,965.16)	€4.62 (- €1,994.52 - €1,799.57)	€3,58 (- €1,663.48 - €5,466.16)
*ICU*				
Mean [95% CI]	€1,002.09 [€813.30 – €1,190.89]	€1,373.09 [€1,098.05 - €1,648.13]	- €496.91 [- €685.70 – - €308.11]	- €125.91 [- €400.95 - €149.13]
Median (Range)	€883.11 (- €92.31 - €2,484.94)	€1,275.75 (- €65.70 - €2,577.90)	- €615.89 (- €1,591.31 - €985.94)	- €223.25 (- €1,564.70 - €1,078.90)

*CI* Confidence Interval, *GIT* gastrointestinal tract, *GW* general ward, *ICU* intensive care unit, *VAS* vascular artery surgery

### Subgroup GIT/GW

For scenario A, average cost savings of €1,512.98 [95% CI: €1,261.29 – €1,764.67] were found within a margin of - €495.52 (aG-DRG G26B^1^) to €3,298.57 (aG-DRG G03A^2^), depending on the invoiced aG-DRG. The median was similar at €1,503.62. For scenario B, average savings of €13.98 [95% CI: - €237.71 - €265.67] were found. The mean was lower at €4.62, and the simulated aG-DRG-remuneration decreased ranging from - €1,994.52 (aG-DRG G26B^1^) to €1,799.57 (aG-DRG G03A^²^).

### Subgroup VAS/GW

Average cost savings were highest within subgroup VAS/GW for scenario A. An average of €1,611.28 [95% CI: €1,016.58 - €2,205.98] was saved per case with a slightly lower median of €1,502.58. Cost-saving results per aG-DRG were ranging from - €164.48 (aG-DRG T60D^3^) to €6,965.16 (aG-DRG F06A^4^). Simulated cost savings for the application of two NGS tests led to average savings of €112,28 [95% CI: - €482,42 - €706,98] and a median of €3,58 within the range of - €1,663.60 (aG-DRG T60D³) to €5,466.16 (aG-DRG F06A^4^).

### Subgroup GIT/ICU

By reducing the LOS of GIT/ICU by four days, average cost savings of €1,002.09 [95% CI: €813.30 – €1,190.89] were found for scenario A. Median savings were slightly lower at €883.11. The range for cost savings extended from - €92.31 (aG-DRG A11H^5^) to €2,484.94 (aG-DRG A07A^6^). For scenario B, a negative budget impact was found with a mean of - €496,91 [95% CI: - €685.70 – - €308.11] and a median of - €615.89. Simulated cost savings per aG-DRG ranged from - €1,591.31 (aG-DRG A11H^5^) to €985.94 (aG-DRG A07A^6^).

### Subgroup VAS/ICU

**Fig. 2:**
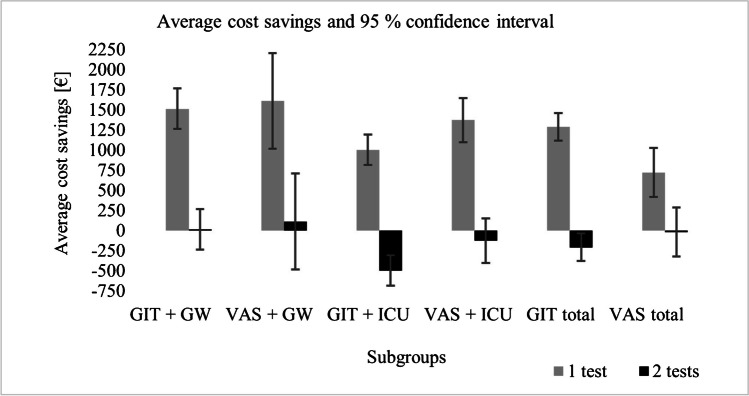
Average cost saving and 95% confidence interval per case for each subgroup. GIT: gastrointestinal tract, GW: general ward, ICU: intensive care unit, VAS: vascular artery surgery

Results of VAS/ICU showed average cost savings of €1,373.09 [95% CI: €1,098.05 - €1,648.13] for scenario A with a range of - €65.70 (aG-DRG E40C^7^) to €2,577.90 (aG-DRG A09A^8^), whereas the median was slightly lower at €1,275.75. Average cost savings for scenario B were negative, resulting in mean additional costs of €125.91 [95% CI: - €400.95 - €149.13] per case. The range was located between - €1,564.70 (aG-DRG E40C^7^) and €1,078.90 (aG-DRG A09A^8^) with a negative median of - €223.25. The results of the budget impact calculations for one and two NGS test(s) are depicted in Fig. 2.

### Break-even analysis

The break-even analysis ‘test cost’ showed that within a two-test scenario, a reduction in total test costs of €95.42 equaling to reduced unit costs of €47,71 per test would achieve a neutral result from a hospital’s perspective. Thus, for a neutral result at the administration of two DISQVER® tests each test would have to cost €1,451.29, compared to the current €1,499. The second analysis ‘test number’ showed that a minimum positive contribution margin can be achieved with an average number of 1.936 tests per case across the whole study population. Further, the break-even analysis of GIT revealed that the break-even point would be reached at 1.9 tests, for VAS at 2.09 tests, per case for €1,499 per test compared to the cost savings due to reduced LOS.

### Statistical analysis

**Fig. 3:**
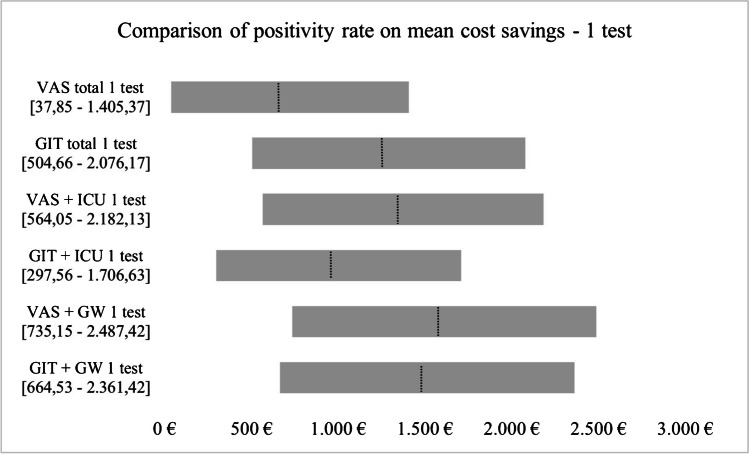
Comparison of the positivity rate on mean saved costs – 1 test

**Fig. 4:**
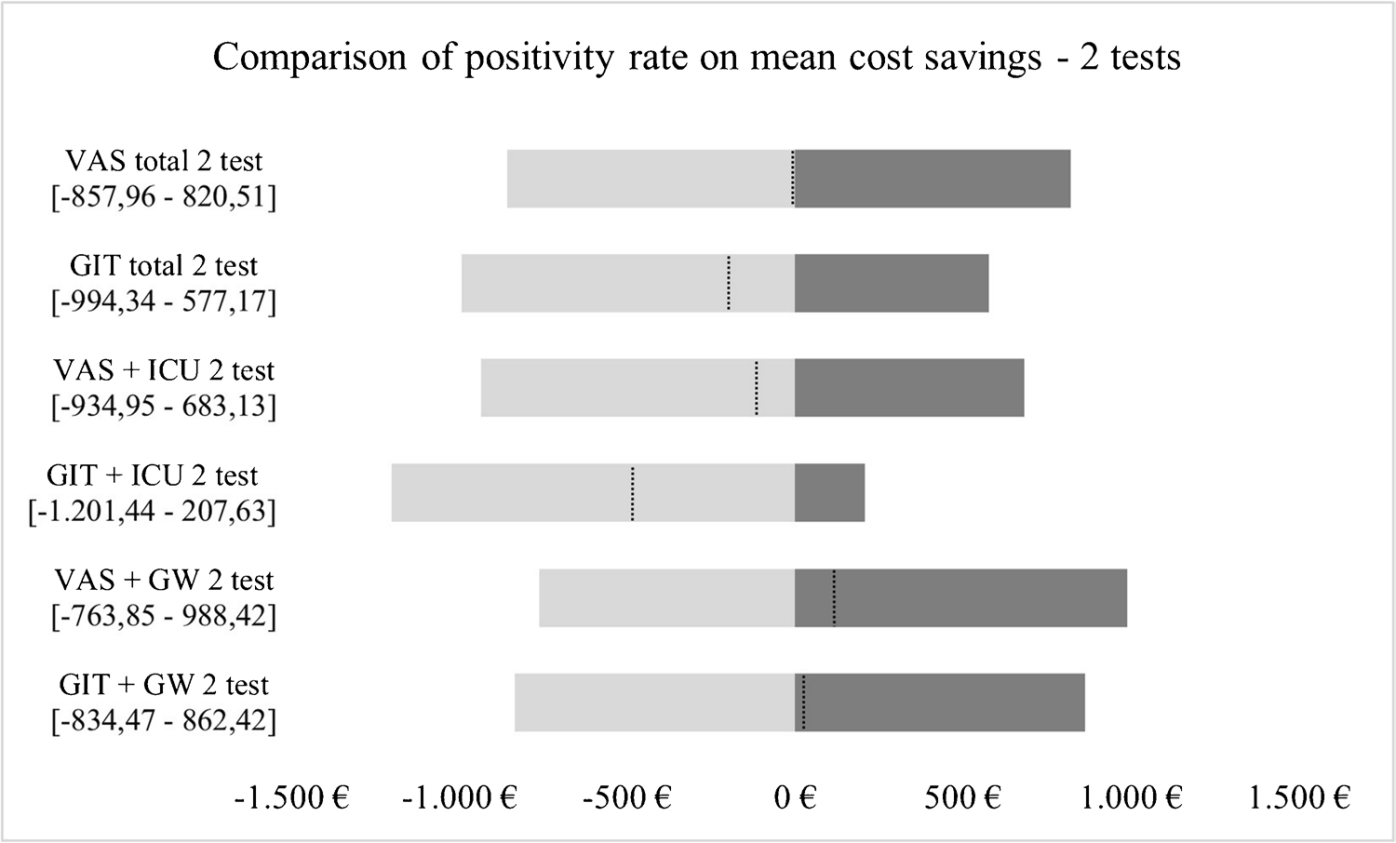
Comparison of the positivity rate on mean saved costs – 2 tests

The results of the deterministic sensitivity analysis are presented in Figs. 3 and 4. The tornado diagram for one test shows that mean cost savings are more influenced by 91% than 51% positivity rate and subgroup VAS was most robust to changes. The tornado diagram for two tests shows that mean saved costs for GW are influenced greater by a 91% positivity rate, whereas all remaining subgroups were rather influenced by a 20% reduction in positivity rate.

## Discussion

To date, this study is the first to analyse the budget impact and break-even point of the pathogen test DISQVER® compared to the current standard procedure of blood culture from a healthcare provider’s perspective in Germany. Based on the assumptions of Grumaz et al. (2019), the use of NGS can reduce inpatients’ LOS resulting in potential cost savings for hospitals. Cost savings were measured based on the costs per day saved according to the aG-DRG flat rate [15].

The budget impact was positive for all subgroups when one NGS test was used. Solely cases of the ICU averaged to a negative simulated budget impact when two NGS tests were issued per case. Across all subgroups, the highest cost savings were found in cases with particularly high case severity and serious complications, as well as long ventilation durations and complex treatments. With an average CMI of 10.453 and an above-average LOS of 43.22 days per case across the entire study population, this study analysed severely ill and multimorbid patients. In comparison, the average LOS typically spans 7.2 days in Germany. Thus, the patient population under study was hospitalized approximately six times longer than the average patient [24]. These findings are consistent with the literature as patients treated in the ICU have an elevated risk of sepsis development attributable to the underlying conditions and invasive and often immunosuppressive treatments [25, 26]. Consequently, generalizing the results to the general patient population would overestimate cost reductions. Although ICU cases are associated with more extensive treatment costs and higher aG-DRG flat rates compared to the GW, the highest average simulated cost savings were found in GIT + VAS for one NGS test. This effect could be attributed to the twofold reduction of LOS for GW cases compared to ICU cases based on Grumaz et al. (2019) [15].

Potential cost savings for GW cases exhibit a broad range. This is particularly evident within subgroup VAS/GW, indicating a wide spectrum of aG-DRG-fees (range - €164.48 - €6,965.16 for one performed test). However, lower outliers generally denote cases with a comparatively low aG-DRG-remuneration leading to potential additional costs of up to €495.52 for GIT and €164.48 for VAS per case for one NGS test in the GW. Upper outliers would generate substantial potential cost savings of up to €6,965.16 in the VAS group for scenario A, though being an outlier. The large variation in potential cost savings observed across different subgroups reflects the heterogeneous aG-DRG fees of this study population. This fluctuation suggests that financial implications of NGS tests can vary significantly depending on the case severity and patient population. Some cases would substantially benefit from NGS testing in terms of cost savings, whereas additional costs would incur for others. Therefore, medical personnel should always consider the need and potential benefits for each case before utilizing one or several NGS tests. Moreover, German hospitals must act in accordance with the efficiency principle according to section 12 of the code of social law which stipulates the assessment of the application of a medication or procedure on its sufficiency, appropriateness, and efficiency [27]. Under this law and based on the findings of this study, NGS should be considered an additional diagnostic test for severe, urgent, and resource-intensive cases rather than an extensive replacement of blood culture tests [28]. From an economic point of view, it seems sensible to narrow down the cases for which NGS tests are used at an early stage.

As of this study’s publication, no economic analysis of NGS for sepsis was identified to verify the results. Nonetheless, Torchia et al. (2019) conducted a cost-effectiveness analysis on NGS vs culture-based methods for diagnosing periprosthetic joint infection (PJI) after total knee arthroplasty suggesting that NGS is cost-effective for diagnosing PJI after a sufficiently high pretest probability (48.3% or greater) [29]. Tan et al. (2017) conducted a systematic review of the cost-effectiveness of NGS in cancer management to test the impact of NGS on cancer diagnosis and patient management. Although they found NGS to be clinically effective at identifying mutations, the six studies varied widely regarding test and treatment costs yielding mixed results. They highlighted the lack of evidence and the need for further research regarding the cost-effectiveness [30]. Moreover, concerns regarding the reimbursement of NGS testing costs were raised as a barrier to its widespread implementation [30]. These findings are in line with the results of this budget impact analysis. The break-even analysis revealed that at the current test costs of €1,499 (€2,998 for two) a reduction by €47.75 per test (€95.50 for two) would on average suffice for costs to be compensated by simulated cost savings. The inclusion of DISQVER® tests in the reimbursement system in Germany through additional reimbursement options beyond the aG-DRG reimbursement fee would lower or avoid the financial risk for healthcare providers and enable sufficient and appropriate care for patients.

Sub-group analyses revealed a positive budget impact for one NGS test per case. Moreover, the findings suggest that potential reductions in resource consumption are closely linked to the type and severity of the inpatient cases as well as the number of pathogen tests required. Nevertheless, this study was based on a retrospective comparative study based on aggregated hospital data [15]. To validate the impact of NGS tests on LOS and cost reduction, further prospective research is necessary. Cost analyses from a micro-costing perspective or modelling of indirect cost savings by avoiding negative outcomes would allow for a more detailed analysis beyond the mere calculation of aG-DRG remunerations. Additionally, improved patient outcomes, quality of life, and reduced need for long-term care are further benefits that can be derived from rapid and sensitive pathogen detection methods such as the DISQVER® test, even for economic purposes.

### Limitations

Even though best practice approaches for budget impact analyses were followed according to Sullivan, et al. (2014) [31], following limitations must be considered: First, the publicly available data set used in this study, is comprised of aggregated information. Therefore, average values were used for all analysed parameters. More precise analyses would be feasible with billing data from hospitals. However, this information is not publicly available on a nationwide scale. Second, even though obvious duplicates were removed based on primary and secondary diagnosis, duplicate cases in the data set are possible and unavoidable. Cases that underwent several procedures show as one case for each OPS code and cannot be excluded. Moreover, mortality rates of sepsis patients range from 25% − 50% [1, 2] and are not represented in the study population, since aG-DRG description does not consistently allow for identification and exclusion of deceased cases. Therefore, an overestimation of the number of cases is likely. Third, the study notes limitations in assessing real cost savings in hospitals since aG-DRG flat rates are remunerated for defined LOS periods rather than on a daily basis. Also, the aG-DRG-remunerations are mixed calculations reflecting average costs of a case, which in sum will amortize the costs for hospitals. Thus, the calculated daily remuneration fee might not translate to real daily costs of the hospital and the analysis can only indirectly estimate the impact on healthcare providers’ budgets. Fourth, patients who are treated in the ICU are usually transferred from or to the GW within the same hospital stay. The aG-DRG does not represent both stays separately and merely adds up to either an intensive case or a case on the GW. Therefore, a reduction of LOS for ICU patients of four days was used based on Grumaz et al. (2019) [15]. However, the real effect of the combination of ICU and GW is unknown and might exceed four days. Consequently, the budget impact of NGS might be underestimated for ICU cases. Last, how pathogens were determined and thus excluded as specific sepsis (A41.0 – A41.8) is unknown due to the aggregated database. As it is not possible to specify the type of diagnostic test performed by OPS code, this may lead to an overestimation of the effects of NGS.

This study contributes to the economic evaluation of NGS which is still scarce compared to the medical evidence of its effectiveness. Results show that NGS can save costs due to a reduction in LOS and have a positive budget impact when one test is used per case. NGS tests can support the strained healthcare system by enabling the provision of high-quality healthcare while containing costs. Healthcare professionals should evaluate the use of NGS on a case-by-case basis, considering factors such as aG-DRG fees or reimbursement options, patient characteristics, and clinical outcomes.

### Conclusion

This study offers the first budget impact analysis of NGS from a hospital perspective based on aggregated aG-DRG remuneration data of post-operative sepsis cases of the gastrointestinal tract, kidney, and vascular artery surgery in Germany. The utilisation of one NGS test per case led to a positive budget impact and decreases to a slightly negative budget impact if two tests are applied. A break-even analysis revealed that a test cost reduction of €47.75 would lead to a neutral budget impact. Despite limitations, this study provides valuable insight into economic implications and advantages of NGS for healthcare providers and suggests its implementation as an additional diagnostic tool for severe and urgent cases. Further research is necessary to validate the findings and determine the full economic impact of DISQVER®.

## Data Availability

The data set consists of public data which hospitals report quarterly to the Institute for Hospital Remuneration System (InEK), accessible via the InEK Data Browser [https://datenbrowser.inek.org/DRG202301]. The database provides anonymous, aggregated case information of all German hospitals according to the outsourced German Diagnosis Related Groups system (aG-DRG). For reasons of data protection, cases with a prevalence of less than four are not displayed. The latest complete annual data from January 1st until December 31st, 2022, was extracted and analysed.

## Footnotes

1 G26B: Other procedures on the anus or anoproctoplasty and reconstruction of anus and sphincter for anal fissures and haemorrhoids, age > 15 years, except for specific malignant neoplasm or inflammatory bowel disease, without minor rectal surgery

2 G03A: Major procedures on the stomach, oesophagus, and duodenum or certain procedures on the small and large intestine or on the stomach, oesophagus and duodenum with complex procedure with highly complex intervention or complex intensive care treatment > - / 368 / - expense points

3 T60D: Sepsis with other complication constellation, except in the case of organ transplantation, without complex diagnosis, without extremely severe CC, without complex intensive care treatment > 196 / 184 / 368 effort points or age < 10 years

4 F06A: Coronary bypass surgery with certain multiple complex OR procedures, with complicating constellation or carotid intervention or complex intensive care treatment > 392 / 368 / - effort points

5 A11H: Ventilation > 249 hours, without complex or specific OR procedure, without complex intensive care treatment > 588 / 828/ 1104 points, without complicating constellation, age > 15 years, without complex diagnosis procedure, without extremely severe CC

6 A07A: Ventilation > 999 hours or > 499 hours with intensive care complex treatment > 4900 / 4600 / 4600 effort points, with complex OR procedure or polytrauma and complex intensive care treatment > 3920 / 3680 / 3680 effort points of with highly complex or three-stage intervention

7 E40C: Diseases and disorders of the respiratory organs with ventilation > 24 hours, more than 2 days of occupancy, with complex procedure, without extremely severe CC, except for para- / tetraplegia

8 A09A: Ventilation > 499 hours or < 249 hours with complex intensive care treatment > 2352 / 1932 / 2208 points, with highly complex intervention or complex OR procedure, age < 16 years, with complex intensive care treatment > 1764 / 1932 / - points or with very complex intervention and complex intensive care treatment > - / 2208 / - points
